# Detecting Remote Evolutionary Relationships among Proteins by Large-Scale Semantic Embedding

**DOI:** 10.1371/journal.pcbi.1001047

**Published:** 2011-01-27

**Authors:** Iain Melvin, Jason Weston, William Stafford Noble, Christina Leslie

**Affiliations:** 1 NEC Laboratories America, Princeton, New Jersey, United States of America; 2 Google, New York, New York, United States of America; 3 Department of Genome Sciences, University of Washington, Seattle, Washington, United States of America; 4 Computational Biology Program, Memorial Sloan-Kettering Cancer Center, New York, New York, United States of America; Stanford University, United States of America

## Abstract

Virtually every molecular biologist has searched a protein or DNA sequence database to find sequences that are evolutionarily related to a given query. Pairwise sequence comparison methods—i.e., measures of similarity between query and target sequences—provide the engine for sequence database search and have been the subject of 30 years of computational research. For the difficult problem of detecting remote evolutionary relationships between protein sequences, the most successful pairwise comparison methods involve building *local* models (e.g., profile hidden Markov models) of protein sequences. However, recent work in massive data domains like web search and natural language processing demonstrate the advantage of exploiting the *global* structure of the data space. Motivated by this work, we present a large-scale algorithm called ProtEmbed, which learns an embedding of protein sequences into a low-dimensional “semantic space.” Evolutionarily related proteins are embedded in close proximity, and additional pieces of evidence, such as 3D structural similarity or class labels, can be incorporated into the learning process. We find that ProtEmbed achieves superior accuracy to widely used pairwise sequence methods like PSI-BLAST and HHSearch for remote homology detection; it also outperforms our previous RankProp algorithm, which incorporates global structure in the form of a protein similarity network. Finally, the ProtEmbed embedding space can be visualized, both at the global level and local to a given query, yielding intuition about the structure of protein sequence space.

## Author Summary

Searching a protein or DNA sequence database to find sequences that are evolutionarily related to a query is one of the foundational problems in computational biology. These database searches rely on pairwise comparisons of sequence similarity between the query and targets, but despite years of method refinements, pairwise comparisons still often fail to detect more distantly related targets. In this study, we adapt recent work from natural language processing to exploit the global structure of the data space in this detection problem. In particular, we borrow the idea of a semantic embedding, where by training on a large text data set, one learns an embedding of words into a low-dimensional semantic space such that words embedded close to each other are likely to be semantically related. We present the ProtEmbed algorithm, which learns an embedding of protein sequences into a semantic space where evolutionarily-related proteins are embedded in close proximity. The flexible training algorithm allows additional pieces of evidence, such as 3D structural information, to be incorporated in the learning process and enables ProtEmbed to achieve state-of-the-art performance for the task of detecting targets that have remote evolutionary relationships to the query.

## Introduction

Using sequence similarity between proteins to detect evolutionary relationships—protein homology detection—is one of the most fundamental and longest studied problems in computational biology. A protein's function is strongly correlated with its 3D structure, and due to evolutionary pressure, protein structures diverge much more slowly than primary sequences. Because protein sequence data will always be far more abundant than high-quality 3D structural data, the computational challenge is to infer evolutionarily conserved structure and function from subtle sequence similarities. When the evolutionary distance is large and the sequence signal faint—so-called *remote homology detection*—this problem is still unsolved.

Stated in purely computational terms, remote homology detection involves searching a protein database for sequences that are evolutionarily related (even remotely) to a given query sequence. Most work in this area has focused on developing more sensitive pairwise comparisons between the query and target sequences, including sequence-sequence local alignments (BLAST [1], Smith-Waterman [2]); profile-sequence (PSI-BLAST [3]) and HMM-sequence comparisons (HMMER [4]); and, most recently, profile-profile [5] and HMM-HMM (HHPred/HHSearch [6]) comparisons. From a machine learning point of view, these recent methods involve building a model of the *neighborhood* of the query and of the target in protein sequence space and using the local neighborhood models to compute a better similarity measure. However, recent advances in massive data domains such as web search and natural language processing suggest that the *global* structure of the data space can also be exploited. For example, motivated by the success of Google's PageRank algorithm, we previously developed RankProp [7], an algorithm that uses graph diffusion on the *protein similarity network*, defined on a large protein sequence database, in order to re-rank target sequences relative to the query and substantially improve remote homology detection.

In the current study, we are motivated by large-scale learning of language models in recent work in natural language processing (NLP) [8]. This NLP work exploited large online text data sets (e.g., Wikipedia) to learn an *embedding* of words into a low-dimensional semantic space, inducing an embedding of sentence fragments. The embedding algorithm iteratively pushes pairs of real sentence fragments together and pulls pairs of real and randomized sentence fragments apart. Thus, at the end of training, words that are near each other in the embedding space are likely to be semantically related. Moreover, the embedding representation can be leveraged to simultaneously train models to solve multiple NLP tasks, using the framework of multitask learning [9].

Here, we present an algorithm called ProtEmbed that learns an embedding of protein domain sequences into a semantic space such that proximity in the embedding space captures homology relationships. After this large-scale training procedure, remote homologs of a query sequence can be detected by mapping the query to the embedding space and retrieving its nearest neighbors. Furthermore, as in the NLP case, we can use multitask learning to incorporate auxiliary information, where available, to improve the embedding, including structural class labels from databases such as SCOP [10] or structural similarity scores for pairs of training examples where both 3D structures are known. It is important to note that our embedding is defined naturally on protein domain sequences rather than multidomain sequences. In particular, inclusion of multidomain sequences in the training data can lead to incompatible distance relationships in the semantic space due to lack of transitivity, resulting in a worse embedding. At testing time, it may be possible to resolve the domain structure of a multidomain query sequence using the learned embedding (see Discussion); however, we only evaluate performance on domain sequence queries in the current study.

We show that ProtEmbed achieves state-of-the-art performance for remote protein homology detection, outperforming our previous algorithm RankProp, which also exploits global structure but uses a *fixed* weighted similarity network rather than a *learned* embedding. Our procedure also yields statistical confidence estimates and enables a visualization of the learned protein embedding space, giving new intuition about the global structure of the protein sequence space.

## Methods

### Semantic protein indexing

The main idea of our approach is to learn a mapping of protein domain sequences into a vector space that captures their “semantic similarity”, i.e. closeness in the semantic space should reflect homology relationships between sequences.

In order to learn an embedding of protein sequences into a semantic space, we need to define (i) a feature representation for proteins, (ii) a training signal that determines whether a given pair of training sequences are similar and should be pushed together by the algorithm, or dissimilar and should be pulled apart, and (iii) an algorithm that learns an appropriate embedding.

Let us denote the set of proteins in the database as 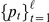 and a query protein as 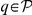, where 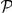 is the set of all possible sequences of amino acids. We then choose a feature map 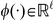 to represent proteins as vectors. This map is necessary so that we can perform geometric operations on proteins. We use the following representation for a protein 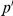:
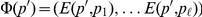
where 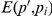 is the E-value returned by a surrogate protein alignment algorithm, such as PSI-BLAST, suitably transformed. Following Rankprop [7], we use the following transformation:
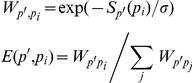
where 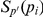 is the PSI-BLAST E-value assigned to protein 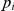 given query 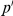 and where we set the parameter 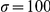. This transformation yields a stochastic connectivity matrix; i.e., the value 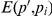 can be interpreted as the probability that a random walk on the protein similarity network will choose to move from protein 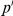 to protein 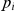. Note that, because most protein pairs exhibit no detectable similarity according to an algorithm such as PSI-BLAST, most feature values are zero. (Specifically, PSI-BLAST assigns a large maximal E-value to all database sequences for which no homology to the query is detected, and the exponential transfer function converts these values to zero.) The sparseness of the feature vectors will be important for computational reasons.

Next, we again use a surrogate protein alignment algorithm, this time as a *teacher* to provide a noisy training signal. We construct a training set of tuples 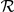, where each tuple contains a query 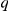, a related protein 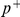 and an unrelated (or lower ranked) protein 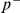. The tuples themselves are collected by running PSI-BLAST in an all-versus-all fashion over the database of proteins. Taking any given protein 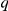 as the query, we consider any protein with an E-value lower than 0.1 to be a similar protein (instance of a 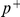); in the current implementation, instances of 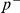 are chosen randomly from all training examples and with high probability will be dissimilar to 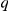. We can then, in principle, construct all possible combinations (tuples) from which we sample randomly during online training.

Given the feature vectors and the training tuples, our aim is to learn a feature embedding that performs well for protein ranking and classification tasks. We will learn an embedding function
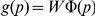
where 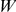 is an 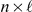 matrix, resulting in an embedding 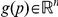. Typically, 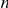 is chosen to be low dimensional, e.g. 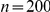. The learning procedure consists of finding a matrix 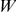 such that similar proteins have close proximity in the embedding space. Specifically, we would like to choose 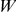 such that, for all tuples 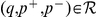,
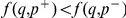
expressing that 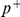 should be ranked higher than 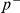, relative to an appropriate distance measure 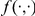 in the embedding space. We define this distance measure using the 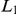-norm (which is defined as 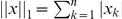):
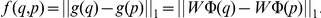



After training, given a query protein 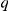, we will rank the database using the ranking score:
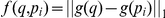
where we consider smaller values of 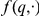 to be more highly ranked.

The training objective employs the margin ranking loss [11], which has been used successfully in the field of information retrieval to rank documents given a query [12]–[14]. That is, we minimize:
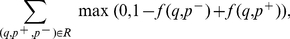
(1)which encourages 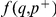 to be smaller than 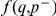 until a margin constraint of 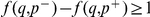 is satisfied. Intuitively, the algorithm tries to push 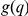 and 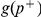 together while pulling 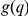 and 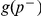 apart, until the difference in distances achieves a margin of 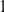. For an equivalent formulation, we can introduce a slack variable 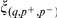 for each tuple 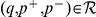 and enforce the constraints
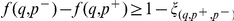
for all tuples while minimizing the objective function
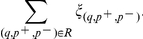



This optimization problem is solved using stochastic gradient descent [13]: iteratively, one picks a random tuple 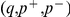 and, if 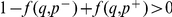, makes a gradient step for that tuple as follows:
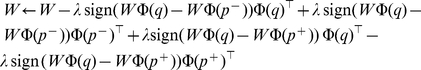
(2)where 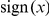 denotes that the sign function is applied componentwise to the vector 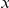 to yield a vector of 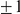 values. Pseudocode for training the ProtEmbed embedding is given in Algorithm 1 in Text S1.

One can exploit the sparsity of 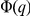 and 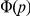 when calculating these updates to make them computationally cheap. To train our model, we choose the (fixed) learning rate 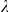 that minimizes the training error, i.e. the loss defined by equation (1). We initialize the matrix 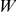 randomly using a normal distribution with mean zero and standard deviation one. Overall, stochastic training is highly scalable and is easy to implement for our model, and learning can scale to millions of proteins.

After training, we precompute the embedding 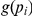 for every protein in the database. At test time, given a query protein 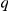, we compute its linear embedding once. Then we are left with only 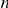 operations per protein in the database to perform when retrieving results for that query.

### Adding information about protein structure

In general, recognizing remote homology relationships among protein structures is easier than recognizing remote homologies based only on protein sequences. Although structural information is available for only a subset of the proteins in the database, we would like to ensure that our embedding captures this structural information in addition to the sequence-based information provided by PSI-BLAST. We consider two sources of structural information: (1) category labels for a given protein and (2) similarity scores between pairs of proteins. For the the category labels, we use the Structural Classification of Proteins (SCOP) [10]. For pairwise similarity scores, we use pairwise structure alignments of known 3D structures using MAMMOTH [15].

We incorporate this auxiliary information using the framework of multitask learning: in addition to the main embedding task, we simultaneously learn models to solve additional tasks using appropriate subsets of the training data. The tasks share internal representations learned by the algorithm, in this case, the embedding function 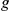. In particular, we pose an auxiliary classification task using SCOP categories, and we pose an auxiliary ranking task using either SCOP category relationships or using MAMMOTH similarities. In all cases, the multitask objective function is simply the sum of the original ProtEmbed objective function and of that of the auxiliary task. We consider these two task types in turn.

**Class-based data.** For auxiliary data in the form of a class label 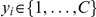 for protein 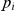 we train an auxiliary classification task that is multitasked with the original ProtEmbed objective, sharing the same embedding space. For each fold and superfamily class we create a vector 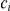, 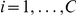, which can be thought of as a set of class centroids. We then would like to satisfy the constraints:
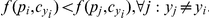
That is, proteins belonging to some class should be closer to that class centroid than proteins that do not belong to that class. We train this model using the margin ranking loss as before, and multitask this problem with the original objective using the following updates:
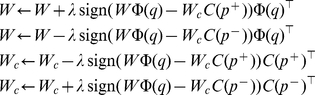
(3)Here 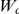 is a matrix containing the centroid vectors 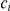 as columns, and 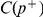 (resp. 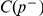) is the bit vector of length 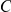 whose two non-zero entries are placed at indices for the fold and superfamily of the labeled training example 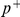 (resp. 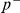). Pseudocode for training the ProtEmbed embedding with class-based auxiliary data is given in Algorithm 2 in Text S1.

**Ranking-based data.** For auxiliary data in the form of similarity scores between pairs of proteins, we simply add more ranking constraints into the set of tuples 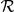. That is, we consider additional tuples of the form 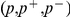 where 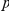 and 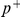 are similar SCOP proteins based on auxiliary data—i.e., a similarity score comparing these proteins is above a cutoff value—while 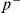 is chosen at random from all of SCOP and with high probability will be structurally dissimilar to 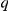. Then we require these additional tuples to satisfy constraints of the form
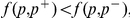
analogous to the constraints in the main optimization problem. Two examples of the use of such auxiliary constraints are given by using SCOP superfamily labels or MAMMOTH. For SCOP labels, if two proteins are in the same superfamily, we say they are similar. For MAMMOTH, we choose a cutoff value of 2.0, and a pair of proteins that has a structural alignment scoring above this cutoff is deemed to be similar. Pseudocode for training the ProtEmbed embedding with ranking-based auxiliary data is given in Algorithm 3 in Text S1.

### Data sets

For labeled data—namely, proteins with structural category labels and 3D structures from which to compute pairwise similarity scores—we used proteins from the SCOP v1.59 protein database. We used ASTRAL [16] to filter these sequences so that no two sequences share greater than 95% identity. This filtering resulted in 7329 sequences. Our test set consists of 97 proteins selected at random from these SCOP sequences. These test sequences were excluded entirely from the training data.

For unlabeled data, i.e. protein domain sequences without category labels or structural information, we used sequences from the ADDA domain database version 4 [17] (http://ekhidna.biocenter.helsinki.fi/downloads/adda). This database contains 3,854,803 single-domain sequences. We removed from the database sequences comprised entirely of the ambiguity code “X,” sequences shorter than 6 amino acids and sequences longer than 10,000 amino acids. We then randomly selected sequences from the remaining sequences until we had picked 3% of the original sequences. This left us with an unlabeled single domain database of 115,644 sequences.

We ran PSI-BLAST version 2.2.8 on the combined SCOP+ADDA database using the default parameters, allowing a maximum of 6 iterations. For a second and more powerful pairwise sequence similarity method based on HMM-HMM comparisons, we also ran HHSearch version 1.5.0, using default parameters. HHPred/HHSearch is considered a leading method for remote homology detection [6]. When searching for homologs to the test set domains, we added the HHSearch options “-realign -mact 0,” which uses local Viterbi search followed by MAC to realign the proteins globally on a local posterior probability matrix. Similarly, MAMMOTH was run with its default settings.

We first trained embeddings on SCOP+ADDA (with SCOP test sequences held out) using PSI-BLAST or HHSearch as the pairwise sequence comparison method to serve as “teacher” for producing 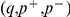 tuples. In this setting, we did not make use of the category labels or structural information for the SCOP training examples. We then trained embeddings using ADDA as unlabeled data and SCOP as labeled data, where the labeled data was used in (i) an auxiliary classification task based on SCOP category labels or (ii) an auxiliary ranking task based either on SCOP category relationships or on MAMMOTH similarity scores.

## Results

### A two-dimensional embedding of proteins

**Figure 1:**
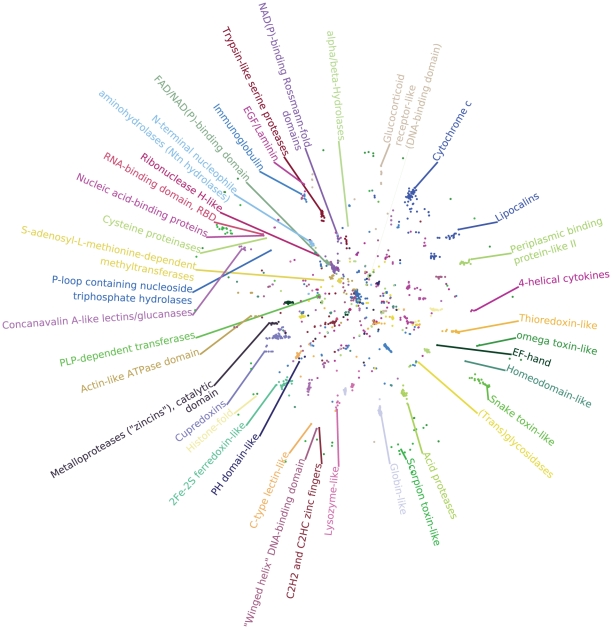
Visualization of the protein embedding. Visualization based on training ProtEmbed with dimension 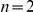 and viewing 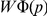 for SCOP proteins from all superfamilies with 25 or more members.

As an initial proof-of-concept test of the ProtEmbed algorithm, we created an embedding of protein domains into a two-dimensional space. This embedding is necessarily underfit, because two dimensions does not provide very much capacity to learn a good embedding. However, a two-dimensional space has the advantage of being easy to visualize. We trained the embedding using the 7329 SCOP proteins from the training set, and then calculated the locations of the all SCOP proteins from all superfamilies with 25 or more members. Figure 1 shows these locations. Proteins are colored and labeled according to their SCOP superfamilies. The embedding generally places members of the same superfamily near one another.

### ProtEmbed provides accurate rankings

To investigate the ability of ProtEmbed to rank homologous proteins above non-homologs, we used a gold standard derived from the SCOP database of protein domain structures. We then used PSI-BLAST, Rankprop, HHSearch and ProtEmbed to rank a collection of 7329 SCOP domain sequences with respect to each of 97 test domains. To provide a rich database in which to perform the search, we augmented the SCOP data set with 115,644 single-domain sequences from the ADDA domain database. In our evaluation, protein domains that reside in the same SCOP superfamily as a query domain are labeled positive, and domains in different folds than that of the query are labeled negative. The remaining sequences—from the same fold but different superfamilies—are ignored, because their homology to the query is uncertain. For each query, traversing the ranked list of labeled sequences induces a receiver operating characteristic (ROC) curve, which plots the percentage of positives as a function of the percentage of negatives observed thus far in the ranked list. We measured the area under this curve up to the first false positive (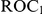) or the 50th false positive (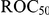). Both scores are normalized such that perfect performance corresponds to a score of 1.0.

Before training our embedding, we ran a series of cross-validation experiments within the training set to select *hyperparameters*; i.e., parameters that are not subject to optimization. Based on these experiments, we used, for PSI-BLAST, a learning rate of 0.05 and an embedding dimension of 250; and for HHSearch, a learning rate of 0.02 and an embedding dimension of 100. In each case, the training was run for 150 epochs, where one epoch corresponds to 20,000 tuples. We used the same hyperparameters when training with or without the auxiliary, structural information.

**Figure 2:**
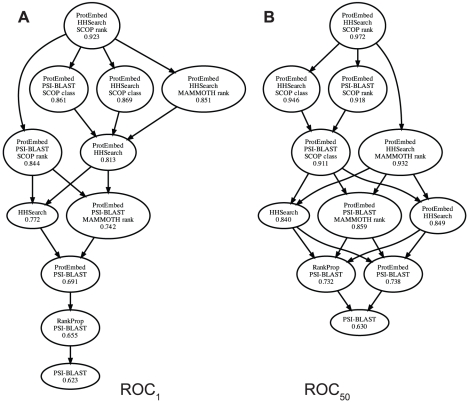
Comparison of mean ROC scores. Each node corresponds to a homology detection algorithm, and the value associated with each node is the mean ROC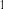 (A) or ROC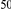 (B) score achieved with respect to 97 test queries. An edge between nodes X and Y indicates that method X performs better than method Y, according to a Wilcoxon signed-rank test with a 0.05 significance threshold.

Figure 2 compares the performance of PSI-BLAST, RankProp, HHSearch and various versions of the ProtEmbed algorithm. The performance of each algorithm is summarized by the mean 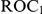 or 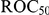 score. To establish the statistical significance of the observed differences, we used a Wilcoxon signed-rank test with a 0.05 significance threshold. For both of the performance metrics that we considered, the ranking of the three previously described methods is the same: HHSearch outperforms Rankprop, which outperforms PSI-BLAST. Also, the standard ProtEmbed algorithm, with no auxiliary data, outperforms PSI-BLAST when it is trained using PSI-BLAST and outperforms HHSearch when it is trained using HHSearch, although for the latter comparison, the difference is only significant for the 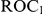 performance metric. Figure 2 in Text S1, which plots the number of queries for which the 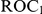 or 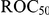 score exceeds a given threshold, shows that the differences among methods are not traceable to queries with particularly high or low ROC values; on the contrary, the improvements from one method to the next span the entire range of ROC values.

Figure 2 shows that adding auxiliary, structural information during ProtEmbed training significantly improves the quality of the resulting rankings. Adding structural information to ProtEmbed improves the mean 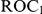 score by 0.038–0.170 and improves the 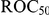 by 0.083–0.180. Perhaps most strikingly, if we consider ProtEmbed trained from HHSearch, the initial embedding is 0.154 away from a perfect 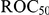 score, whereas the embedding learned using SCOP rankings is only 0.025 away from a perfect 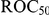 score. Thus, in this case, structural information removes 83.7% of the residual error. In general, using SCOP information leads to better rankings than using MAMMOTH. This is not surprising, because we are using a gold standard based on SCOP. Between the two modes of representation, the SCOP ranking appears to give better results than using SCOP class-based structural information. This result is somewhat surprising, because our gold standard is based explicitly on SCOP classes and perhaps suggests that the ranking representation is more resistant to overfitting.

**Figure 3:**
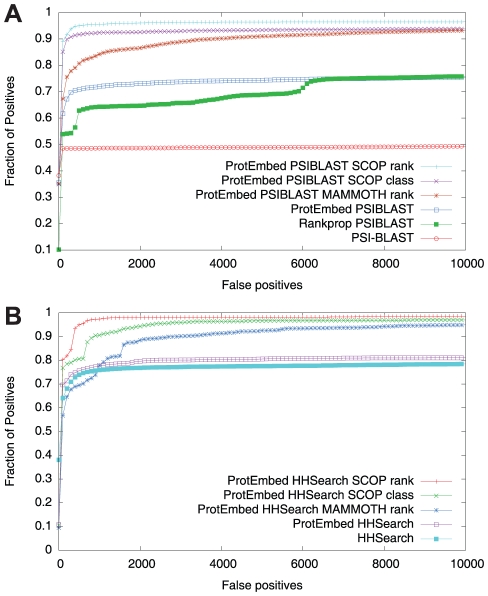
Combined evaluation across multiple queries. Each panel shows a collection of ROC curves, produced by sorting into a single ranked list the results from all 97 test queries. Each series corresponds to a different algorithm. The panel on top (A) includes algorithms based on PSI-BLAST; the panel on the bottom (B) includes algorithms based on HHSearch.

In evaluations of remote homology detection algorithms, some researchers prefer to ignore members of the same family as the query, since these family members are presumably easy to identify [18]. To ensure that our results are not dependent on family-level information, we repeated the ROC calculations above, but we skipped target proteins that fall into the same family as the query. Figure 3 in Text S1 shows that the conclusions above remain unchanged in this setting: ProtEmbed outperforms HHSearch, RankProp and PSI-BLAST, and using structural information significantly improves ProtEmbed's performance.

### Calibration of ProtEmbed scores

Next, we evaluated how well ProtEmbed scores are calibrated between queries. We say that our scores are well calibrated if pairs of query and target sequences at similar distances from each other in embedding space also have similar degrees of homology, regardless of where the query embeds. If this property holds, then the scores generated by ranking database sequences relative to different queries can be compared to each other and modeled to assign statistical significance.

The experiment reported in Figure 2, in which ROC scores are computed separately for each query and then averaged, only measures how well the target sequences in the database are ranked relative to each query sequence. To measure the calibration of the scores among queries, we sorted all of the scores from all 97 test queries into a single list. The resulting ROC curves are shown in Figure 3. The overall ranking of methods is the same as in Figure 2, in order of improving performance: PSI-BLAST, Rankprop, HHSearch, ProtEmbed. To obtain calibrated scores, PSI-BLAST, Rankprop and HHSearch include specific calibration procedures—calculation of E-values for PSI-BLAST and HHSearch, and calculation of superfamily probabilities for Rankprop. ProtEmbed, in contrast, requires no explicit calibration procedure; instead, the scores are naturally calibrated because they all correspond to distances in a single embedding space.

To be useful, a homology detection algorithm must provide scores with well defined semantics. For example, PSI-BLAST reports an expectation value, or E-value, that corresponds to the number of scores as good or better than the observed score that are expected to occur in a random database of the given size [3]. Rankprop reports for each query-target pair the probability that they belong to the same SCOP superfamily [19]. To convert ProtEmbed distances to an interpretable score, we employed a simple empirical null model in which protein sequences are generated by a third-order Markov chain, with parameters derived from the SCOP+ADDA database. We randomly generated decoy protein sequences according to this null model, and we embedded these proteins into the PSI-BLAST ProtEmbed space. Empirical analysis of the resulting sets of scores (Figure 1 in Text S1) shows that the left tail of the null distribution is well approximated by a Weibull distribution. To compute a *p*-value, we select the null distribution based on the length of the given query sequence. Further details are given in Text S1.

We cannot use these *p*-values directly, because we must correct for the large number of tests involved in searching a large sequence database. To do so, we employ standard false discovery rate-based multiple testing correction procedures. In particular, for a given query, we first estimate the percentage 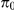 of the observed scores that are drawn according to the null distribution [20]. We then use the Benjamin-Hochberg procedure [21] to estimate false discovery rates, including the multiplicative factor 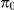. Finally, we convert the estimated false discovery rate into a *q*-value [20], which is defined as the minimum FDR threshold at which an observed score is deemed significant.

### Visualizing the results of a query

For many users of alignment tools such as PSI-BLAST, the multiple alignment produced with respect to a given query is as useful as the rankings and accompanying E-values, because the multiple alignment provides an explanation of the ranking. However, a method like ProtEmbed does not rely solely on multiple alignments. Therefore, although it would certainly be feasible to create, in a *post hoc* fashion, an alignment of the ranked proteins up to, e.g., a specified ProtEmbed *q*-value threshold, such a multiple alignment is not likely to accurately reflect the semantics of the ProtEmbed embedding space. Instead, we propose to use a multidimensional scaling approach to project the top-ranked protein domains into an easy-to-visualize 2D representation.

To illustrate how effective such a visualization can be, we systematically generated 2D maps of the neighborhood for all 97 test set domains, using a *q*-value threshold of 0.01. Thumbnail versions of all 97 neighborhoods are provided in the supplement.

**Figure 4:**
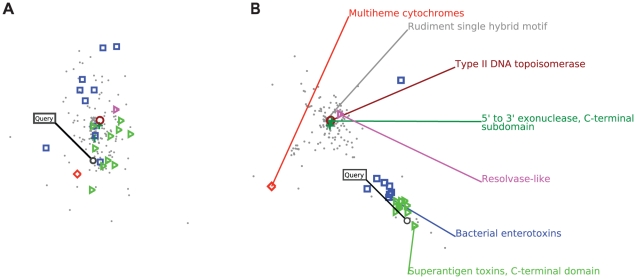
Neighborhood of a query in the embedding space. (A) To visualize the effect of the embedding, we first show a query, the C-terminal domain of Staphylococcal enterotoxin B (PDB ID 3seb), mapped into a metric space according to the PSI-BLAST based feature representation used to initialize the embedding algorithm. (B) The query is now mapped into the final embedding space. In both panels, the query is labeled and indicated with a black circle. All members of the same SCOP family (superantigen toxins, C-terminal domain; SCOP 1.75 ID d.15.6.1), indicated with green triangles, are now in a tight cluster around the query and disambiguated from a distinct but functionally related SCOP superfamily (bacterial enterotoxins; SCOP 1.75 ID b.40.2), indicated with blue squares. Unrelated superfamilies are well separated from the query in the embedding space; members of unrelated SCOP superfamilies are indicated by various colored shapes, as labeled in the right panel.

Here, we focus on a single example. Figure 4 shows the structure learned by the embedding near a particular query, the C-terminal domain of Staphylococcal enterotoxin B (PDB ID 3seb). Figure 4(A) shows the neighborhood of the query relative to the initial PSI-BLAST based feature embedding of the domain sequences, projected into 2D for easier visualization. This mapping corresponds to the initialization of the embedding algorithm, before any training. We see that the other members of the query's family—the superantigen toxins, C-terminal domain (SCOP 1.75 ID d.15.6.1), shown in green—are generally near the query in the initial embedding, but these true positives are intermingled with members of a functionally related but structurally distinct superfamily, the bacterial enterotoxins (SCOP 1.75 ID b.40.2, shown in blue) as well as several members of unrelated superfamilies. When we map the query sequence into the final embedding space (Figure 4(B)), we now find that it lands in a tight cluster of its family members, which is near but separated from the cluster of related bacterial enterotoxins. Meanwhile, unrelated superfamilies are appropriately separated into distinct clusters distant from the query. In this example, the homology detection performance improves from an 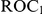 score of 0.091 (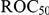 of 0.716) relative to the initial embedding to a perfect 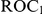 (and perfect 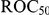) of 1.0 after training.

## Discussion

We have shown that ProtEmbed *learns* an embedding of protein domain sequences such that proximity in the embedding space reflects homology relationships. Due to efficient stochastic gradient descent methods, the training algorithm can scale to millions of sequences. A flexible multitask framework also enables the use of additional label or ranking information, e.g. protein structural classes or pairwise structural similarity scores, where known, to improve the embedding. Given a test query sequence, its embedding can be computed in the same time that it takes to run the underlying pairwise sequence alignment method. The query's homologs can then be efficiently retrieved by determining the nearby database proteins based on their precomputed embedding coordinates. Moreover, using a faster but less accurate pairwise alignment method, such as PSI-BLAST, together with ProtEmbed, when supplied with labeled data through an auxiliary task, leads to better performance than state-of-the-art but slower pairwise alignments methods, such as HHSearch, used on their own. Moreover, use of more sensitive PSI-BLAST parameters rather than the default choices could potentially further improve the performance of the embedding.

While alignment-based pairwise sequence similarity scores are used as features for calculating the embedding, ProtEmbed does not produce multiple sequence alignments for query sequences as an output of its computation. Instead, the embedding neighborhood of the query can be visualized for insight into the relationship between the query and its homologs. For further sequence-based analysis of query-homolog similarities, hits from the ProtEmbed neighborhood could be used to compute an alignment using standard methods [22] or newer graph algorithm approaches [23].

The ProtEmbed algorithm learns its embedding on domain sequences rather than full-length protein sequences, because the embedding only makes sense when transitivity relationships hold. For example, a multidomain sequence will have sequence similarity to its constituent domains, which will typically also be represented as entries in the database; if these domains are dissimilar from each other, then the set of pairwise relationships lead to conflicting constraints during training. Nonetheless, it is possible to process a multidomain query sequences using ProtEmbed by first applying an existing domain decomposition algorithm [24] and then embedding each domain separately. Alternatively, one could potentially use the embedding to help resolve the domain structure: first, one could run a pairwise alignment method such as PSI-BLAST to determine the start and end positions of all the hits, and then these subsequences could be embedded separately as candidate domain sequences. The *p*-value for the score between the embedded candidate sequence and its nearest neighbor in the database should generally favor candidates with boundaries similar to those of the true domains.

Protein sequence analysis is one of the oldest subfields of computational biology, with mature and specialized tools designed to describe the *local* structure of protein sequence space. By adapting new techniques from massive data domains such as natural language processing and web search, we have demonstrated that the *global* structural of protein sequence space can be exploited for classical problems like homology detection.

## Supporting Information

Text S1 Supplementary methods and results.
(1.69 MB PDF)

## References

[1] Altschul SF, Gish W, Miller W, Myers EW, Lipman DJ (1990). A basic local alignment search tool.. J Mol Biol.

[2] Smith T, Waterman M (1981). Identification of common molecular subsequences.. J Mol Biol.

[3] Altschul SF, Madden TL, Schaffer AA, Zhang J, Zhang Z (1997). Gapped BLAST and PSI-BLAST: A new generation of protein database search programs.. Nucleic Acids Res.

[4] Eddy SR, Rawlings C (1995). Multiple alignment using hidden Markov models.. Proceedings of the Third International Conference on Intelligent Systems for Molecular Biology.

[5] Rychlewski L, Jaroszewski L, Li W, Godzik A (2000). Comparison of sequence profiles: Strategies for structural predictions using sequence information.. Protein Sci.

[6] Soding J, Biegert A, Lupas AN (2005). The HHpred interactive server for protein homology detection and structure prediction.. Nucleic Acids Res.

[7] Weston J, Elisseeff A, Zhou D, Leslie C, Noble WS (2004). Protein ranking: From local to global structure in the protein similarity network.. Proc Natl Acad Sci U S A.

[8] Bai B, Weston J, Grangier D, Collobert R, Sadamasa K, Bengio Y, Schuurmans D, Lafferty J, Williams CKI, Culotta A (2009). Polynomial semantic indexing.. Advances in Neural Information Processing Systems 22.

[9] Collobert R, Weston J (2008). A unified architecture for natural language processing: deep neural networks with multitask learning.. Proceedings of the 25th International Conference on Machine Learning.

[10] Murzin AG, Brenner SE, Hubbard T, Chothia C (1995). SCOP: A structural classification of proteins database for the investigation of sequences and structures.. J Mol Biol.

[11] Herbrich R, Graepel T, Obermayer K, Smola, Bartlett, Schoelkopf, Schuurmans (2000). Large margin rank boundaries for ordinal regression.. Advances in Large Margin Classifiers.

[12] Joachims T (2002). Optimizing search engines using clickthrough data.. Proceedings of the Eighth ACM SIGKDD International Conference on Knowledge Discovery and Data Mining.

[13] Burges C, Shaked T, Renshaw E, Lazier A, Deeds M (2005). Learning to rank using gradient descent.. Proceedings of the 22nd International Conference on Machine Learning.

[14] Grangier D, Bengio S (2005). Inferring document similarity from hyperlinks.. Proceedings of the 14th ACM International Conference on Information and Knowledge Management.

[15] Ortiz AR, Strauss CEM, Olmea O (2002). MAMMOTH (Matching molecular models obtained from theory): An automated method for model comparison.. Protein Sci.

[16] Brenner SE, Koehl P, Levitt M (2000). The ASTRAL compendium for sequence and structure analysis.. Nucleic Acids Res.

[17] Heger A, Wilton CA, Sivakumar A, Holm L (2005). ADDA: a domain database with global coverage of the protein universe.. Nucleic Acids Res.

[18] Jaakkola T, Diekhans M, Haussler D (1999). Using the Fisher kernel method to detect remote protein homologies.. Proceedings of the Seventh International Conference on Intelligent Systems for Molecular Biology.

[19] Melvin I, Weston J, Leslie CS, Noble WS (2009). RANKPROP: a web server for protein remote homology detection.. Bioinformatics.

[20] Storey JD (2002). A direct approach to false discovery rates.. J R Stat Soc Series B.

[21] Benjamini Y, Hochberg Y (1995). Controlling the false discovery rate: a practical and powerful approach to multiple testing.. J R Stat Soc Series B.

[22] Kemena C, Notredame C (2009). Upcoming challenges for multiple sequence alignment methods in the high-throughput era.. Bioinformatics.

[23] Heger A, Mallick S, Wilton CA, Holm L (2007). The global trace graph, a novel paradigm for searching protein sequence databases.. Bioinformatics.

[24] Yeats C, Redfern O, Orengo CA (2010). A fast and automated solution for accurately resolving protein domain architectures.. Bioinformatics.

