# Supplementary material for: Detecting Remote Evolutionary Relationships among Proteins by Large-Scale Semantic Embedding
Source: PLoS Comput Biol. 2011 Jan 27;7(1):e1001047. doi: 10.1371/journal.pcbi.1001047 (PMC3029239; doi:10.1371/journal.pcbi.1001047)
Supplement: Text S1 — Supplementary methods and results. (1.69 MB PDF) [file pcbi.1001047.s001.pdf]

Supplement to "Detecting remote evolutionary relationships among proteins by large-scale semantic embedding"

Iain Melvin[1], Jason Weston[2], William Stafford Noble[3*], Christina Leslie[4†]
[1]NEC Laboratories America, Princeton, NJ
[2]Google, New York, NY
[3]Department of Genome Sciences, University of Washington, Seattle, WA
[4]Computational Biology Program, Memorial Sloan-Kettering Cancer Center, New York, NY

Computing q-values using an empirical null model

To construct an empirical null distribution of ProtEmbed scores, we first calculated the parameters of a third-order Markov model from the SCOP+ADDA database. The resulting model was then used to generate a series of 43 sets of 100 sequences, with each set having a different range of allowed lengths. The length ranges are as follows: 10–20, 21–30, 31–40, . . . , 91–100, 101–150, . . . , 951–1000, 1001–1100, . . . , 1901–2000, 2001–2500, . . . , 4501–5000. ProtEmbed was run on each of these 4300 null protein sequences. The database contains 122973 proteins; hence, the calculation of the empirical null yielded a total of over 528 million scores.

Because we are only interested in the smallest ProtEmbed scores, we examined the left tail of the null distribution. Empirically, we observed that the location of the left tail is fairly stable as a function of sequence length, except for a marked shift that occurs around a length of 70 amino acids. This phenomenon is illustrated in Figure 1(A) in this Supplement. For this reason, we partitioned the empirical null distribution into two distributions, corresponding to short (≤ 70 amino acids) and long (> 70 amino acids) sequences, respectively. For each of these two empirical null score distributions, we fit the parameters of a three-parameter Weibull distribution. To do so, we first multiply the scores by -1 and then fit only the right 10% tail of the distribution.

To test the quality of the fit of the Weibull distribution, we randomly divided the null query sequences into a training set and a test set. We learned Weibull parameters from the scores produced by the training set, and then we plotted that distribution with respect to the empirical distribution of scores from the test set. The results, shown in Figure 1 below, show that the Weibull distribution fits the empirical distribution quite well, except in the tail, where we observed a few very small p-values. Examining these small p-values, we found that they corresponded to queries that received extremely small PSI-BLAST E-values. Hence, we conclude that, although some of ProtEmbed's p-values are misleadingly small, the effect is restricted to a small number of queries which occur due to false positive homology relationships identified by PSI-BLAST.

*Co-corresponding author, 1705 NE Pacific Street, Box 355065, Seattle, WA 98195, william-noble@u.washington.edu, Tel: 206-543-8930, Fax: 206-685-7301
†Co-corresponding author, 1275 York Ave, Mail Box #460, New York, NY 10065, cleslie@cbio.mskcc.org, Tel: 646-888-2762, Fax: 646-422-0717

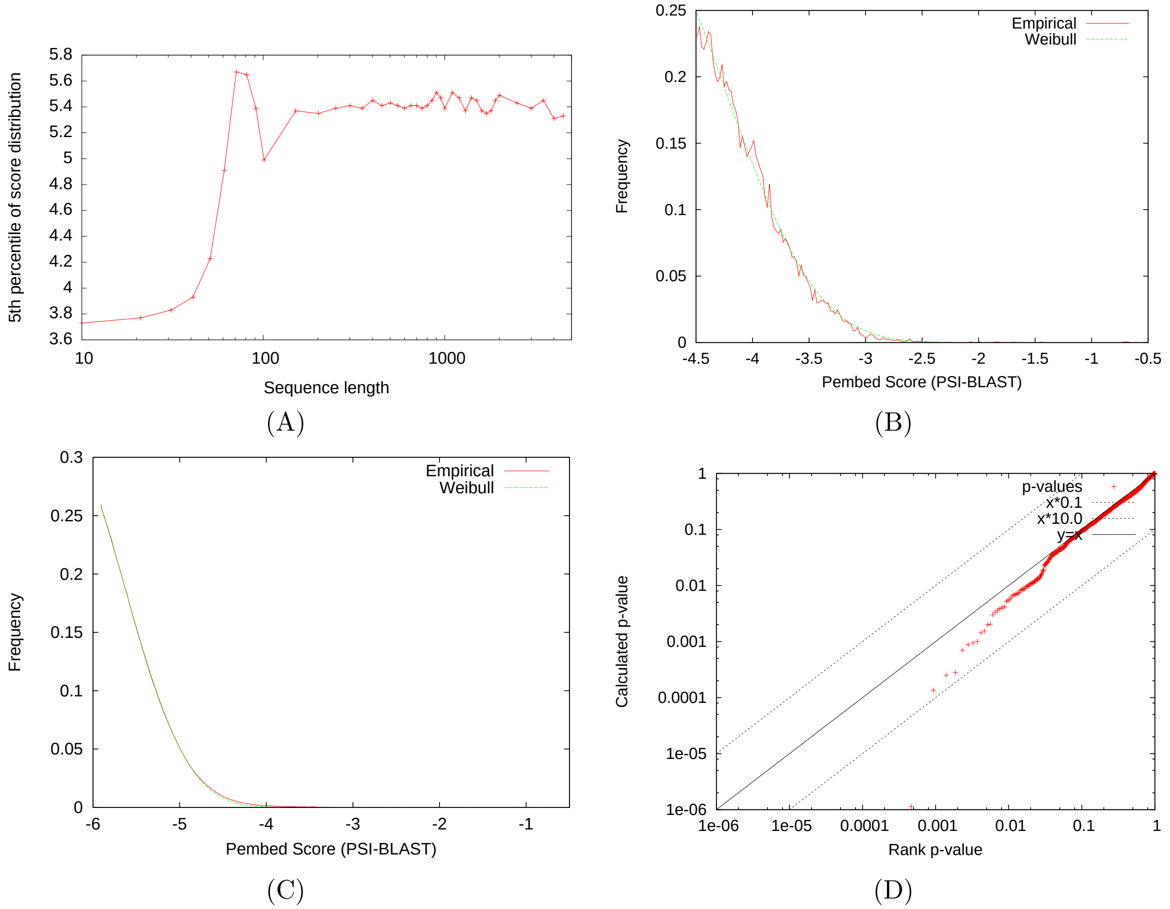

Figure 1: **Fitting the empirical null distribution.** (A) The figure plots the 5th percentile of the empirical null score distribution as a function of sequence length. The left tail shows a marked shift between short ($\leq$ 70 amino acids) and long ($>$ 70 amino acids) sequences. (B) The figure shows the right tail of the (negated) empirical score distribution and the corresponding Weibull distribution, for short ($\leq$ 70 amino acids) sequences. (C) This figure is the same as panel (B), but for long ($>$ 70 amino acids) sequences. (C) This figure plots the calculated p-value as a function of the rank p-value for all sequences in the test set. The rank p-value of a score x is defined as the fraction of scores that are greater than or equal to x. The distribution of p-values is compared against $y = x$ (solid line), $y = 10x$ and $y = x/10$ (dotted lines).

while $i < n$ **do**
  $q \leftarrow$ selectRandom(D)
  $p^+ \leftarrow$ selectRandom(neighborsBelowCutoff(q, t, D));
  $p^- \leftarrow$ selectRandom(D)
  updateGradient(q, p^+, p^-)
  $i \leftarrow i + 1$
end while

Algorithm 1: **ProtEmbed algorithm pseudocode.** The algorithm requires three inputs: the protein domain sequence database D, a threshold t, and the number of iterations n.

while $i < n$ **do**
  $q \leftarrow$ selectRandom(D)
  $p^+ \leftarrow$ selectRandom(neighborsBelowCutoff(q, t, D))
  $p^- \leftarrow$ selectRandom(D)
  updateGradient(q, p^+, p^-)
  $q \leftarrow$ selectRandom(S)
  $p^+ \leftarrow$ selectRandom(getSuperfamily(q, S))
  $p^- \leftarrow$ selectRandom(S)
  updateGradient(q, p^+, p^-)
  $i \leftarrow i + 1$
end while

Algorithm 2: **ProtEmbed+AUXclass algorithm pseudocode.** The algorithm requires four inputs: the protein domain sequence database D, the SCOP structure database S, a threshold t, and the number of iterations n.

while $i < n$ **do**
  $q \leftarrow$ selectRandom(D)
  $p^+ \leftarrow$ selectRandom(neighborsBelowCutoff(q, t, D));
  $p^- \leftarrow$ selectRandom(D)
  updateGradient(q, p^+, p^-)
  $q \leftarrow$ selectRandom(S)
  $l^+ \leftarrow$ getSuperfamilyLabel(q, S));
  repeat
    $l^- \leftarrow$ getSuperfamilyLabel(selectRandom(S), S)
  until $l^+ \neq l^-$
  updateGradient(q, l^+, l^-)
  $i \leftarrow i + 1$
end while

Algorithm 3: **ProtEmbed+AUXrank algorithm pseudocode.** The algorithm requires four inputs: the protein domain sequence database D, the SCOP structure database S, a threshold t, and the number of iterations n.

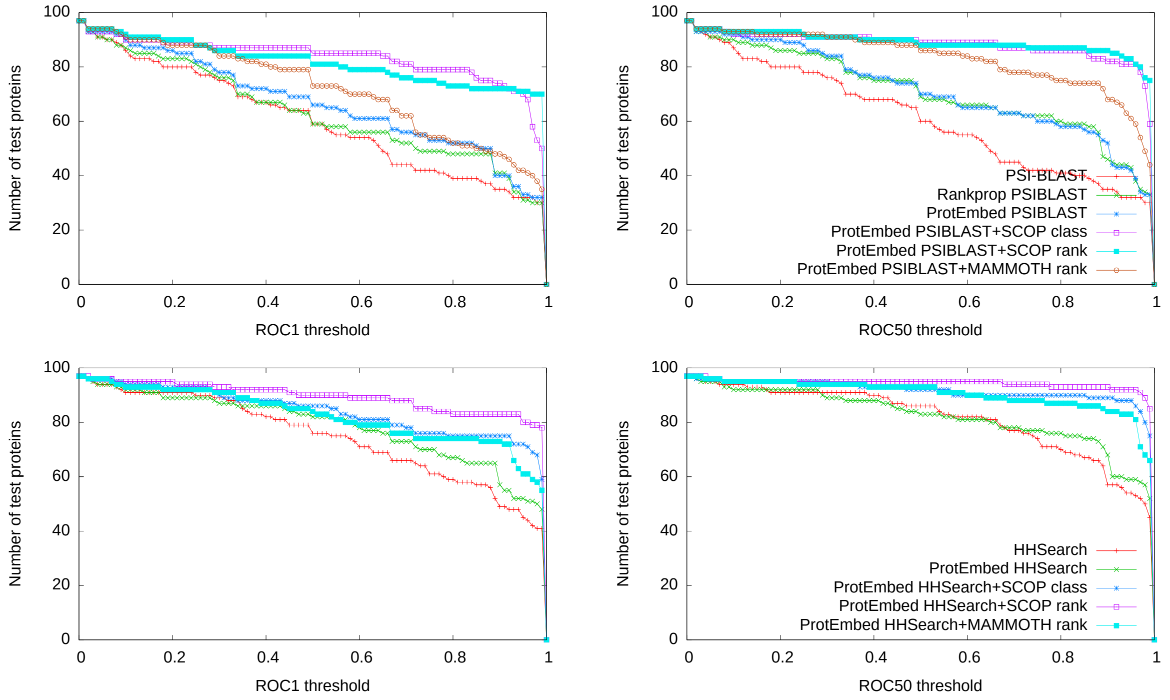

Figure 2: **Comparison of methods.** Each panel plots the number of query sequences (y-axis) whose ROC score exceeds a given value (x-axis). The top two panels compare PSI-BLAST-based methods; the bottom two panels compare HHSearch-based methods. The left two panels use ROC_1 scores; the right two panels use ROC_{50} scores.

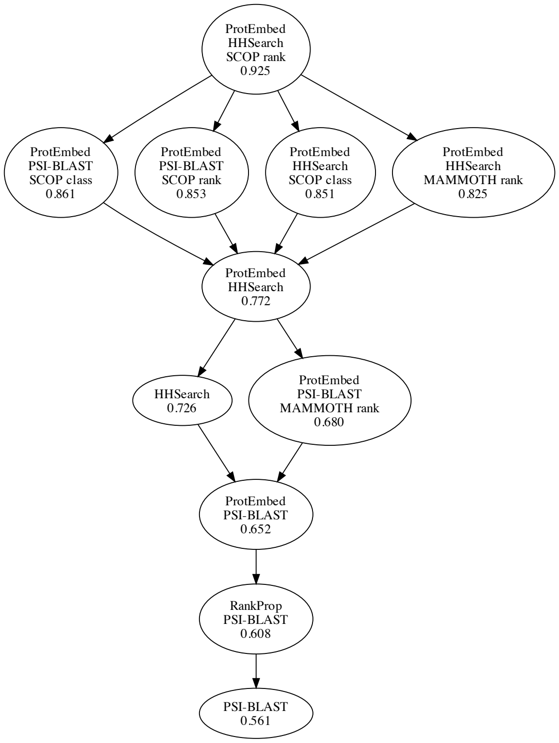

ROC_1

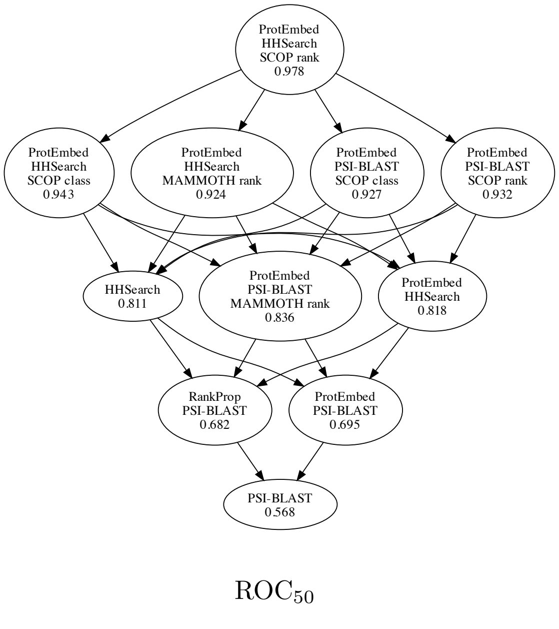

ROC_{50}

Figure 3: **Comparison of methods, ignoring family members.** This figure is similar to Figure 2 in the main text, except that protein domains that reside in the same SCOP family as the query domain are ignored during the ROC calculations.

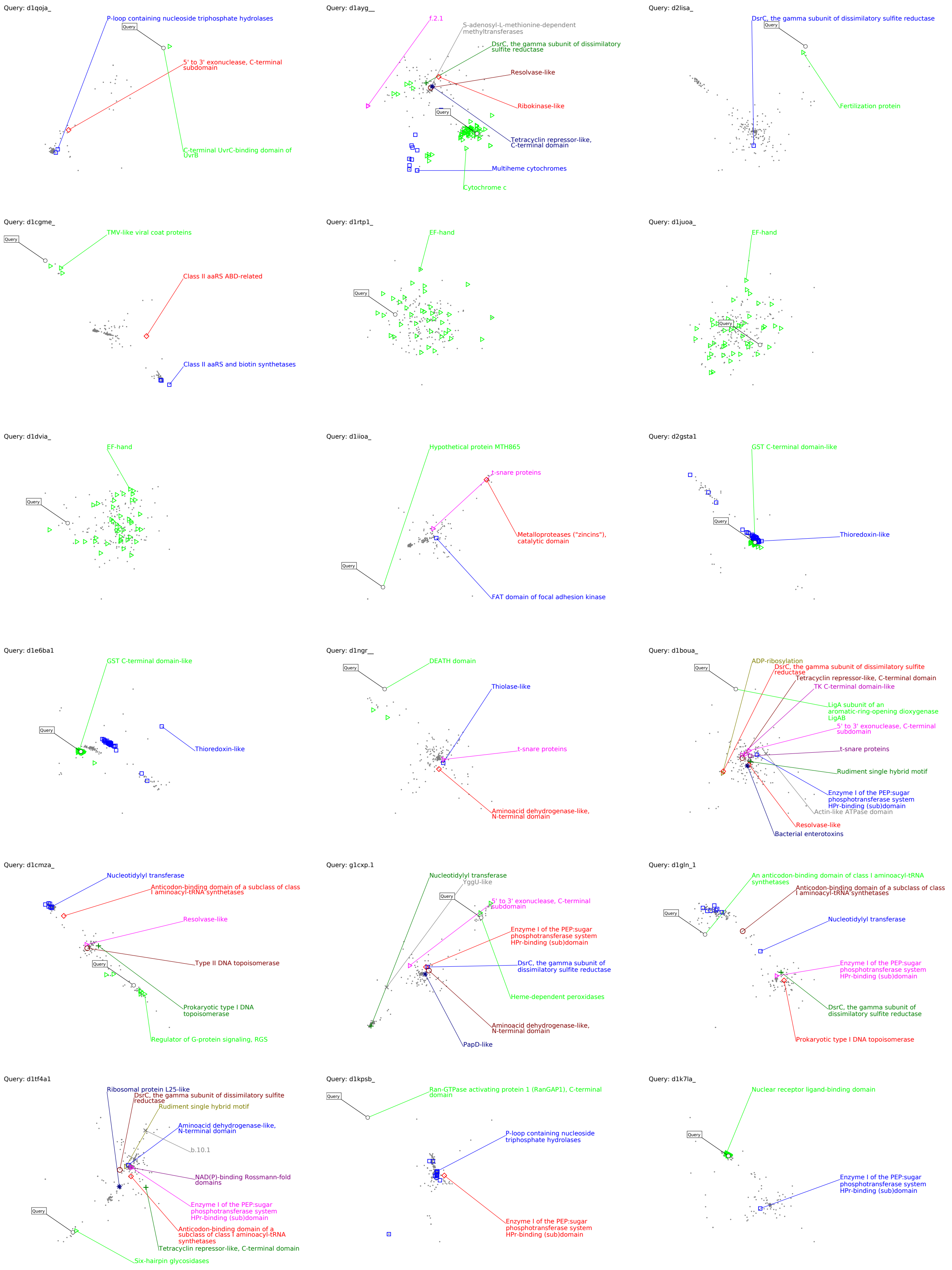

Figure 4: **Visualization of the top-ranked proteins for 97 test set domains.** Queries 1 to 18. Annotations are similar to Figure 4 of main text.

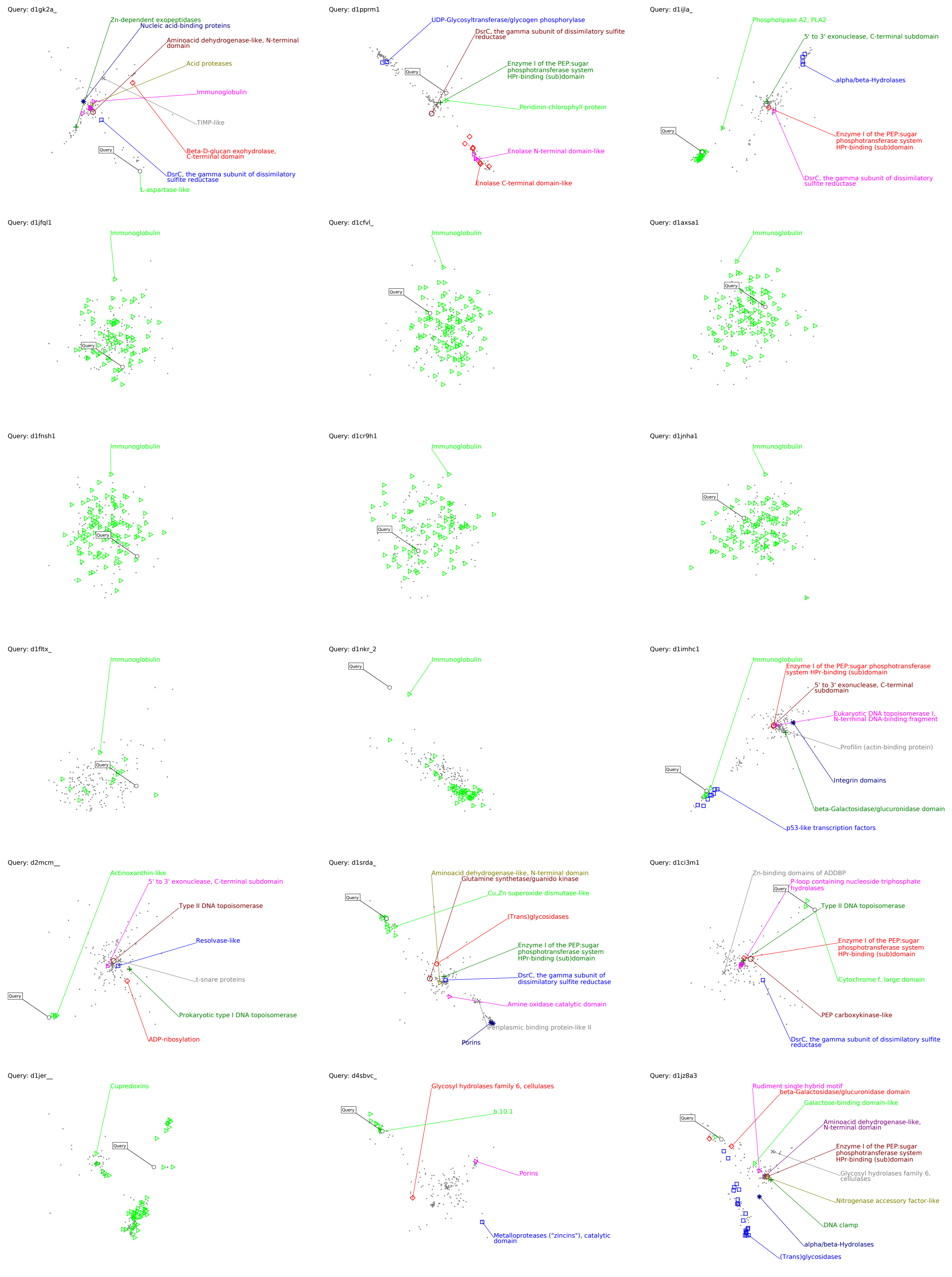

Figure 5: **Visualization of the top-ranked proteins for 97 test set domains.** Queries 19 to 36. Annotations are similar to Figure 4 of main text.

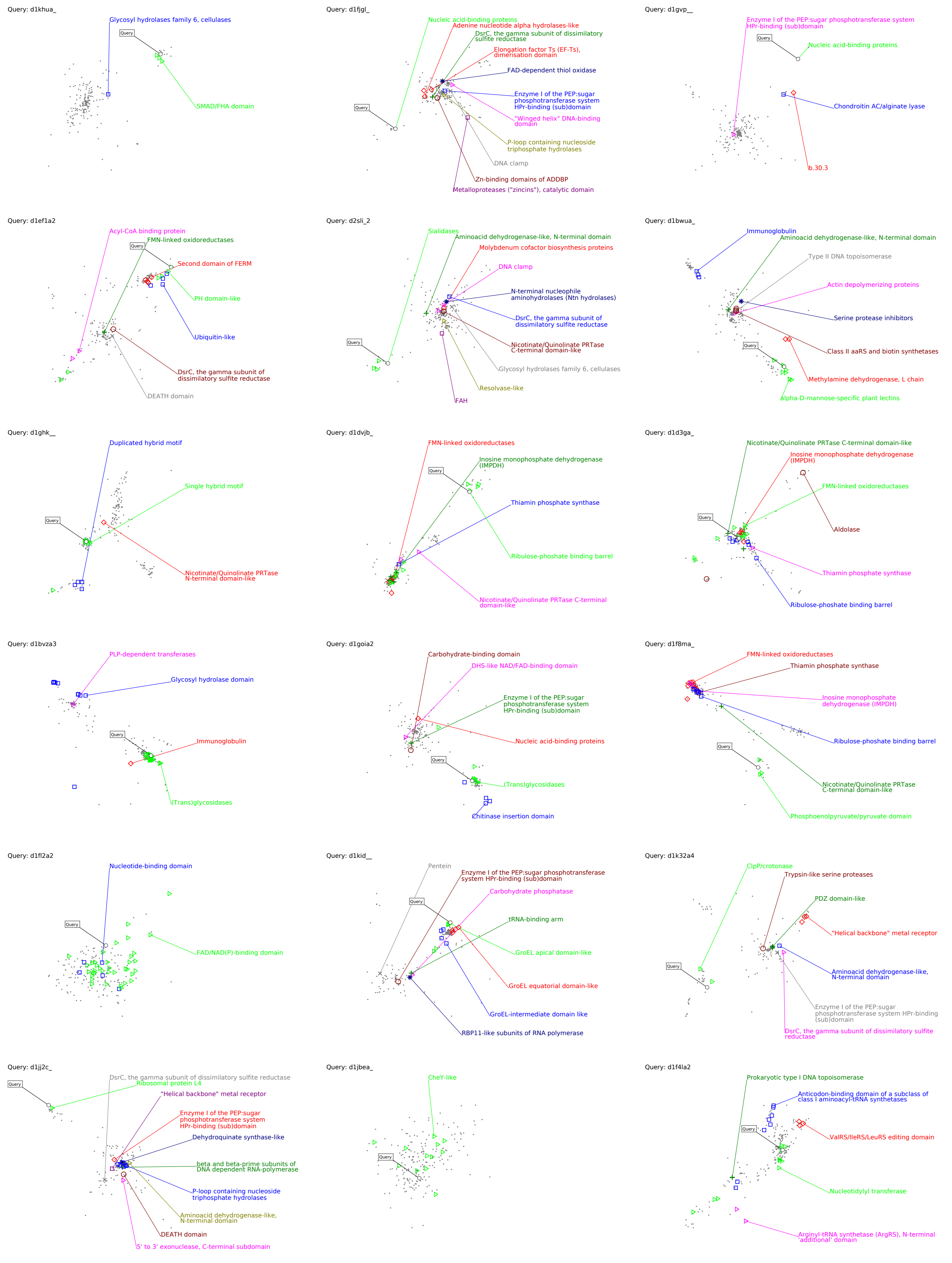

Figure 6: **Visualization of the top-ranked proteins for 97 test set domains.** Queries 37 to 55. Annotations are similar to Figure 4 of main text.

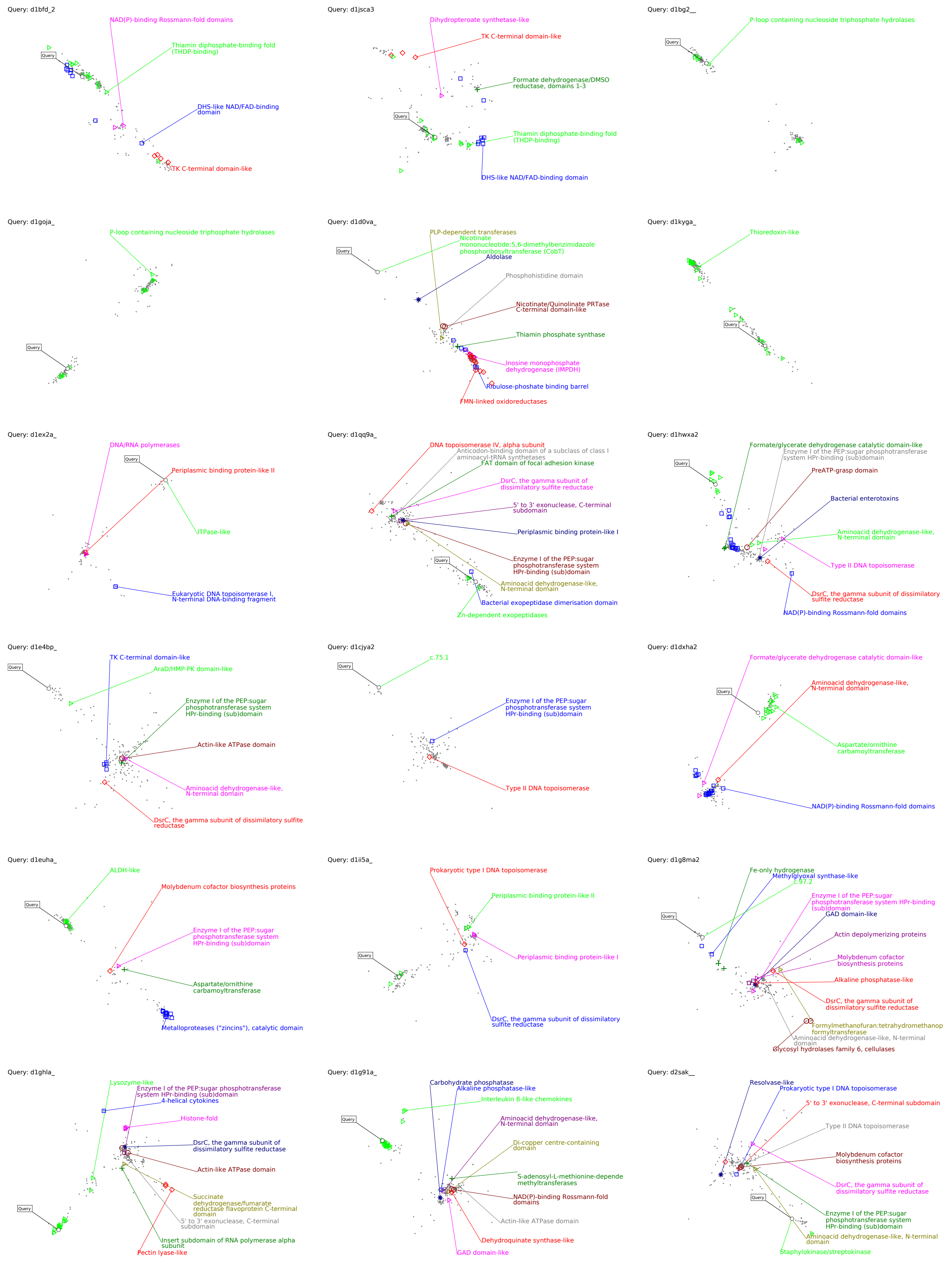

Figure 7: **Visualization of the top-ranked proteins for 97 test set domains.** Queries 56 to 73. Annotations are similar to Figure 4 of main text.

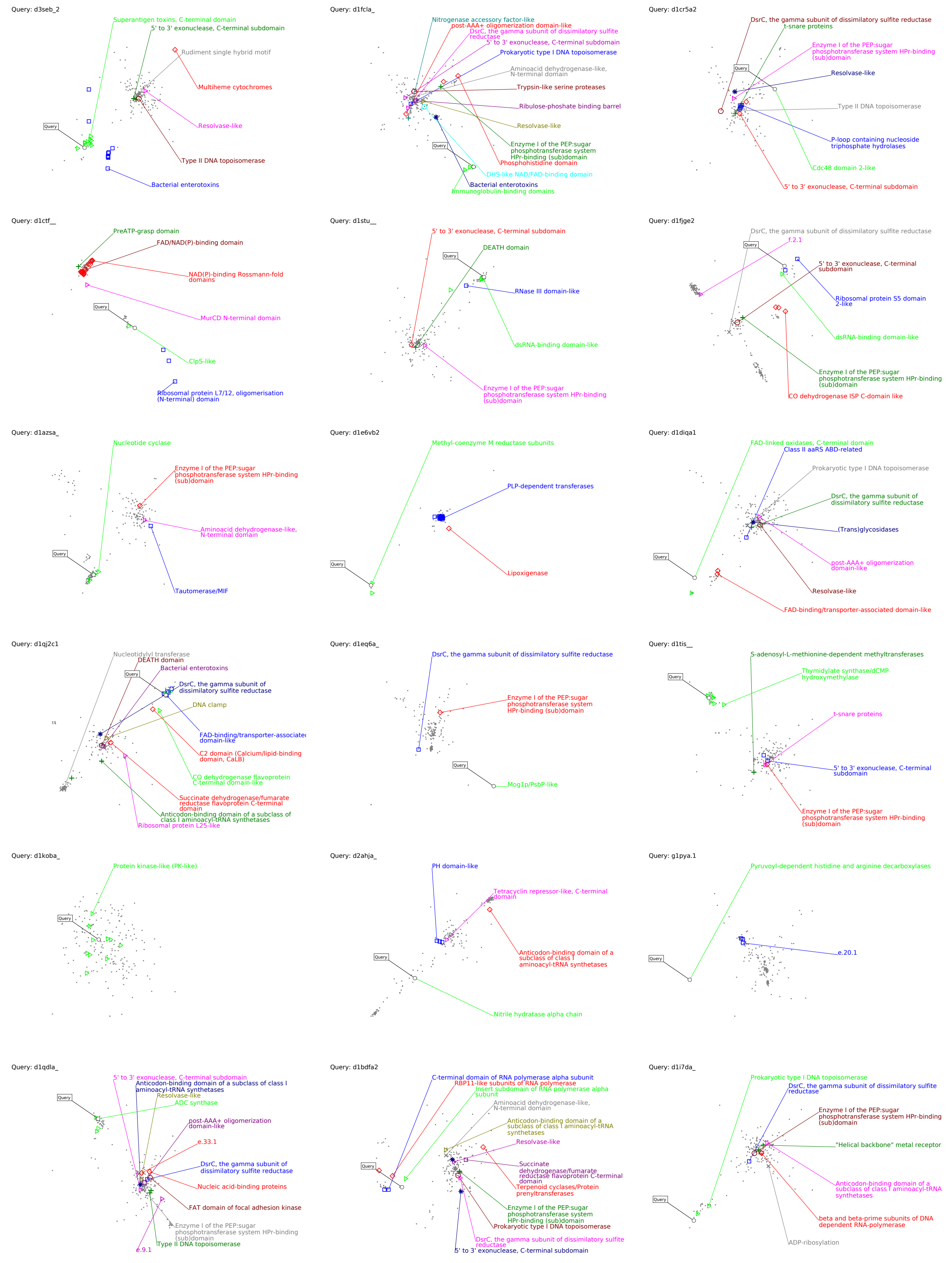

Figure 8: **Visualization of the top-ranked proteins for 97 test set domains.** Queries 74 to 91. Annotations are similar to Figure 4 of main text.

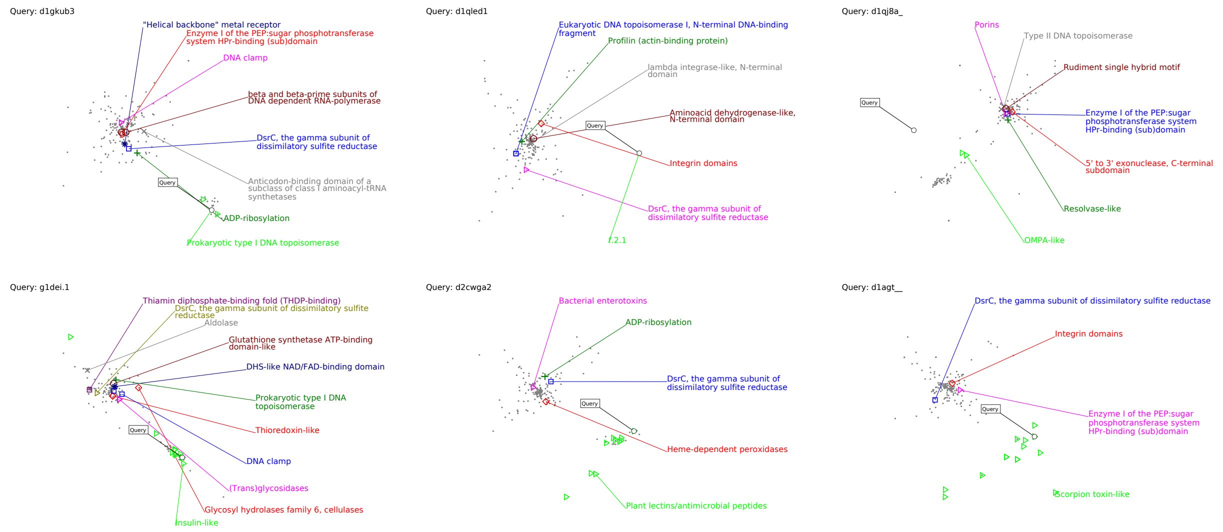

Figure 9: **Visualization of the top-ranked proteins for 97 test set domains.** Queries 92 to 97. Annotations are similar to Figure 4 of main text.
